# Dynamic modeling of TENORM exposure risk during drilling and production

**DOI:** 10.1007/s13202-017-0325-3

**Published:** 2017-02-23

**Authors:** Khalid ALNabhani, Faisal Khan, Ming Yang

**Affiliations:** Centre for Risk, Integrity and Safety Engineering (C-RISE), Faculty of Engineering and Applied Science, Memorial University, St. John’s, NL A1B 3X5 Canada

**Keywords:** TENORM, Occupational risk, Radiation risk assessment, SMART approach, Rational theory

## Abstract

Exposure to Technologically Enhanced Naturally Occurring Nuclear Radioactive Material (TENORM) from oil and gas drilling and production activities can have effects on both the environment and workers involved in the industry. There is a significant lack of available information regarding dynamic modeling and risk assessment of TENORM occupational exposure in the oil and gas industry, and available studies show that workers in the field are at risk of being exposed to varying levels of radiation. This paper presents a methodology to bridge this knowledge gap by modeling workforce TENORM radiation exposure at different oil and gas operation stages. This was achieved by integrating SHIPP (System Hazard Identification, Prediction and Prevention) Methodology And Rational Theory (SMART approach). The SMART approach was applied to develop an integrated framework for TENORM occupational exposure risk assessment. Application of the proposed approach is illustrated with a scenario, and outcomes from modeling this scenario explain how system degraded as a function of safety barrier performance.

## Introduction

Thirty years worth of research has supported the fact that exposure to Technologically Enhanced Naturally Occurring Nuclear Radioactive Materials (TENORM) poses significant risks to people involved in the oil and gas industry (Gesell 1975; Steinhäusler 2005). Regardless of the exposure level, catastrophic cancer could be the eventual consequences of radiation exposure (ALNabhani et al. 2016b). Therefore, accident mitigation involving radiological exposure in the oil and gas industry can be conducted at an early stage through preventative methodologies, including ensuring effective maintenance of appropriate safety measures and barriers, to reduce risk and life-threatening situations. Radiological poisoning from chronic exposure to TENORM is cumulative and thus would be difficult to identify, especially in early stages; therefore, it could take many years for negative health effects to be manifested. The danger of radiation exposure could be combated by periodic medical check-ups for cancer and other negative effects. This situation could be improved by predicting, controlling and mitigating exposure at the source, as well as emphasizing incident prevention to achieve an inherently safer process design to enhance safety. In order to protect health and increase safety by preventing instances of major exposure, it is critical to ascertain the presence and adequacy of safety prevention barriers. The paper focuses on TENORM exposure modeling and risk assessment in typical oil and gas extraction and production operations using the SHIPP (System Hazard Identification, Prediction and Prevention) Methodology And Rational Theory (SMART approach). The proposed approach has the following unique features: (1) dynamic modeling of TENORM occupational exposure considering safety barrier performance, (2) uncertainty reduction throughout prediction of the failure probabilities of safety barriers, and (3) dynamic updating of event probability as new information becomes available. The proposed approach provides an integrated framework for dynamic prediction and TENORM exposure risk information updating. The outcome of this approach would help to monitor radiation exposure risk, support the development of effective safety measures and protective measures and minimize the overall oil and gas operation risk.

## TENORM exposure modeling and risk assessment using the SMART approach

**Fig. 1:**
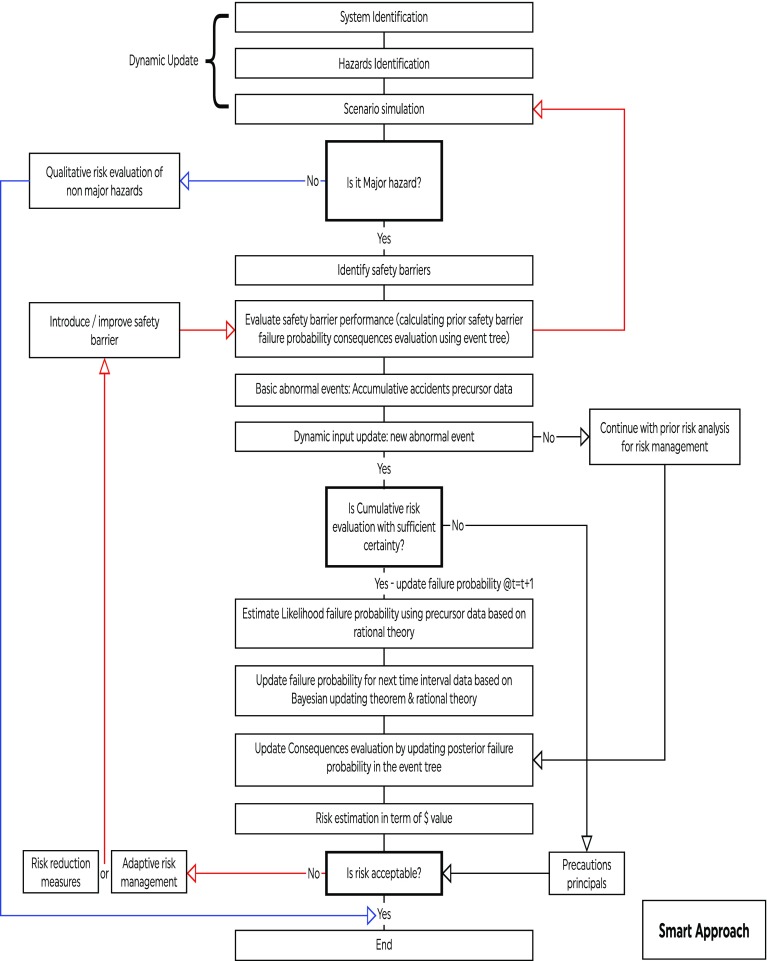
Algorithm of SMART approach

The SMART approach combines the SHIPP methodology and rational theory. The SHIPP methodology is a generic framework used to identify, evaluate and model process accidents (Rathnayakaa et al. 2011). Rational theory is used to model accident causation behavior that usually contributes to its occurrence based on logical, inductive and probabilistic analysis. The basic premise of rational theory is that an accident occurrence is a result of joint conditional behavior among different parameters. By integrating the SHIPP methodology and rational theory, the SMART approach is able to: (1) identify the interaction between systems and their subsystems, the source of TENORM and its distribution in oil and gas extraction and production processes; (2) identify and analyze TENORM exposure scenarios; (3) model different radiation exposure scenarios based on the safety barriers performance using Monte Carlo simulation; (4) predict and update the identified safety barriers failure probabilities; (5) enable proactive management of TENORM risks using either adaptive risk management or precautionary principle methodologies. Figure 1 presents the SMART approach flowchart developed for TENORM occupational exposure risk modeling. The proposed approach was demonstrated and validated using a case study of TENORM occupational exposure scenarios for a 2271 worker sample involved in different kinds of typical oil and gas activities.

## TENORM occupational exposure scenario modeling and prediction

**Fig. 2:**
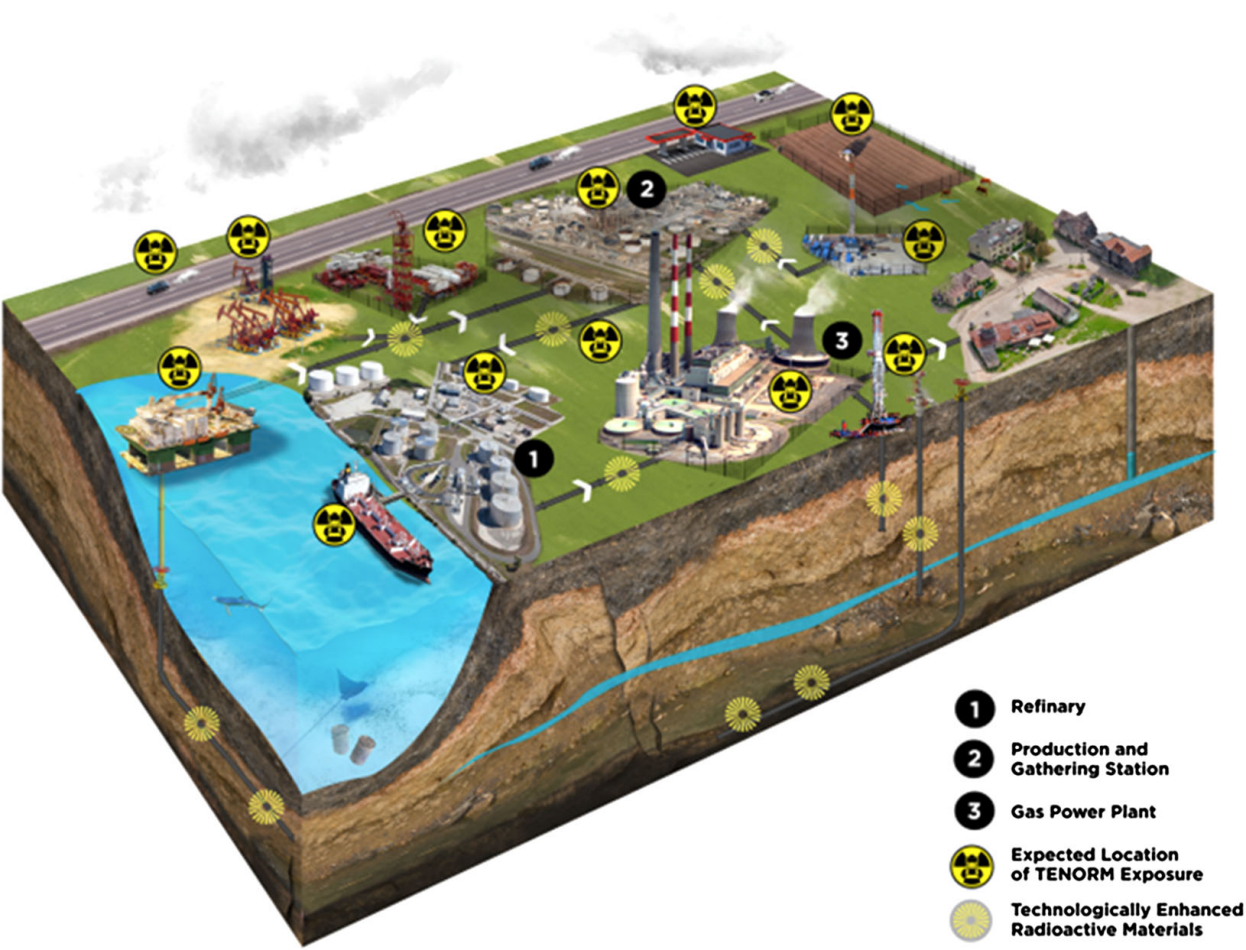
An overview of TENORM presence during oil and gas extraction and production activates

In the present study, scenarios of TENORM occupational exposure were modeled and simulated using the SMART approach. A sample was taken of 2271 workers involved in different oilfield activities, as shown in Fig. 2 in order to simulate different possible radiological occupational exposure in the oil and gas industry as a result of possible failure of identified safety barriers. A period of ten years was considered for serious carcinogenic risk. The prior estimate of abnormal events was used for preliminary decision making, and then the Bayesian theorem was utilized to calculate the posterior failure probabilities of safety barriers during the ensuing time interval, to which the consequence probabilities were generated through an event tree analysis. As new evidence or new information became available at any time during evaluation process, accordingly the safety barrier failure probabilities were updated dynamically. Subsequently, updated risk for each consequence level was estimated using new posterior failure probabilities. This way time-dependent risk profiles were developed dynamically for each TENORM exposure. The intention of the SMART approach was to develop effective risk management strategies to aid in identifying critical safety barriers that need to be maintained in the oil and gas industry and achieve the lowest risk.

### System identification

During oil and gas extraction and production procedures, the oil, gas, formation water and TENORM mixture ascends to the surface via drilled wells through down-hole completion and production equipment. This mixture then travels to midstream equipment via a separator, which removes the gas and relays it to a downstream gas purification plant. The degassed oil stream is further pumped to midstream production from the upstream facilities via flow lines. Gathering and production stations then remove the oily sludge, sand and geological formation water that are contaminated with TENORM. A portion of the TENORM has a solidified form and deposits on the oil field extraction and production equipment internal surfaces (Testa et al. 1994; Kvasnicka 1996; Al-Masri and Aba 2005; Othman et al. 2005; ALNabhani et al. 2015). Pipelines transport crude oil to downstream facilities for further refining, where the refined products may still harbor TENORM. Smith (1992) reported that TENORM can be transported in different forms in the produced hydrocarbons, which confirms their existence wherever there is oil and gas or related products used in power plants, petrochemicals and manufacturing industries. Al-Masri and Haddad (2012) concluded from their study on TENORM emissions from oil- and gas-fired power plants that TENORM was present in fly and bottom ash collected from major Syrian power plants fired by heavy oil and natural gas. On the other hand, many scholars have reported that benzene used in several industry applications found to cause carcinogenic diseases associated with leukemia, and more specifically with acute myeloid leukemia cancer (Vigliani and Saita 1964; Aksoy et al. 1974; Infante et al. 1977; Yin et al. 1978; Jamall and Willhiteb 2008; WHO 2010).

### Safety barriers identification and evaluation

During oil and gas extraction and production processes, five sequential and interconnected safety barriers for radiation prevention could be identified, and they are as follows:

1. Early Detection Safety Prevention Barrier (EDSPB). This is considered to be the release prevention barrier (RPB) that is responsible for preventing the initiating event for TENORM release at the upstream source. This includes, but is not limited to, the following sub-barriers:
  Field and well logging data, such as spectral gamma logs that provide information on early TENORM presence, and its level of radioactivity prediction.
  Down-hole real-time detectors that are capable of detecting the radioactive level from rock formation during drilling activities. Surface sensors should also be fixed at different locations in drilling rigs such as at the cellar, wellhead, flow line connected to bell nipple, mud system, waste pits and rig floor.
  Sensors can be fixed also in flow lines between the wellhead and gathering stations, equipment in the gathering and production stations such as separation tanks and eventually in refinery utilities, particularly in storage tanks.
2. Isolation Integrity Safety Prevention Barrier (IISPB). This is considered to be a dispersion prevention barrier (DPB) at the upstream, midstream and downstream phase. It includes, but is not limited to, the following sub-barriers: equipment insulation carrying TNEROM coproduced with oil and gas, including downhole equipment, wellheads, flow lines, separation tanks, pumps and other associated processing equipment in gathering and production stations; emergency shut-down mechanisms and work permits.
3. Personal Protection Equipment and Exposure Duration Safety Prevention Barrier (PPE&EDSPB). It includes, but is not limited to, the following sub-barriers: disposable leaded shield personal protection equipment (overalls, face mask, hand gloves and safety boots) and personal radiation monitors.
4. Emergency Management Safety Prevention Barrier (EMSPB). This safety barrier is considered as the mitigation barrier to control hazardous TENORM exposure and its consequences. It includes, but is not limited to, the following sub-barriers: emergency response plan, emergency preparedness, emergency medical plan, emergency and safety drills, worker awareness and participation in radiation training programs.
5. Management and Organization Safety Prevention Barrier (M&OSPB). This safety barrier intervenes either positively or negatively with all other barriers based on the management’s behavior and responsibility. It includes, but is not limited to, the following sub-barriers: training programs, safety polices, operating procedures, decision making, management practices and knowledge, leadership and communication.

**Fig. 3:**
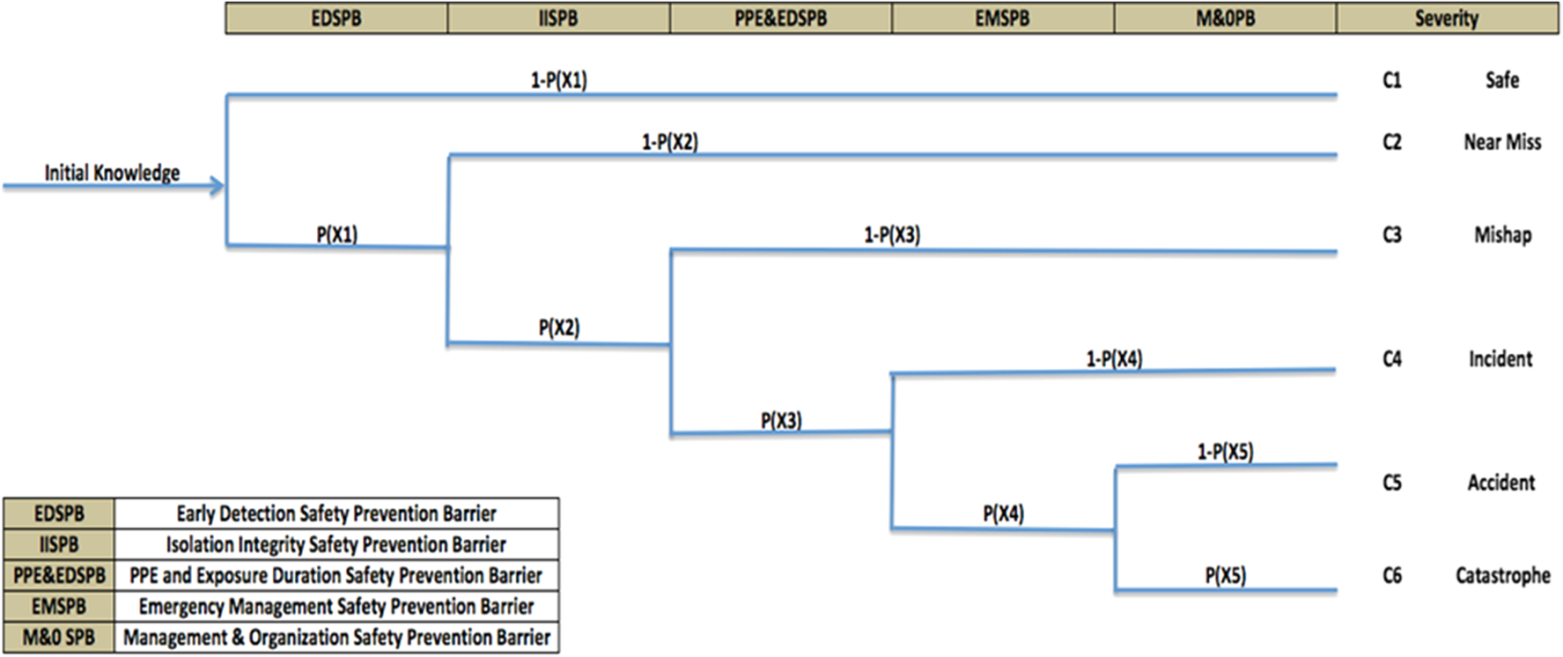
Event tree of TENORM occupational exposure in oil and gas industry

The associated event tree model was utilized to demonstrate the consequences of TENORM exposure based on the failure of each of these identified safety barriers. These five safety barriers were assigned six possible states ranging from safe to catastrophe. The occurrence of each state is possible through failure of different safety barriers, as is shown in Fig. 3.

In this risk assessment, the radiation exposure scenario was described in terms of safety barrier failures. Due to a dearth of relevant literature on this subject, the failure probabilities of the identified safety barriers were assigned by expert judgment with the support of professional academic experts from Centre for Risk, Integrity and Safety Engineering (C-RISE-Memorial University) (Table 1). These values are utilized here for illustration and validation purposes.

**Table 1:** Failure probability of safety barriers (based on expert judgment, C-RISE, Memorial University)

Safety barrier (*X*_*i*_)	Failure probability *P*(*X*_*i*_)
Early Detection Safety Prevention Barrier-EDSPB	0.20
Isolation Integrity Safety Prevention Barrier-IISPB	0.05
Personal Protection Equipment and Exposure Duration Safety Prevention Barrier-PPE&EDSPB	0.05
Emergency Management Safety Prevention Barrier-EMSPB	0.10
Management & Organization Safety Prevention Barrier-M&O SPB	0.10

The failure and success of a safety barrier is represented as node with two outcomes. For example, if first safety barrier EDSPB is successful, then the desirable outcome is “safe.” If it is unsuccessful, the penultimate safety barrier, IISPB, is activated. If this node is successful, the outcome is labeled “near miss.” If unsuccessful, the safety function PPE&EDSPB is activated. The successful outcome of this node is “mishap.” In the case of a failure, then the next safety barrier, EMSPB, is activated which leads to the consequence labeled “Incident.” If this barrier fails, the last safety barrier M&OSPB is activated. When M&OSPB is successful, the end state is labeled “accident.” If M&OSPB is unsuccessful, the end-state consequence is labeled “catastrophe.”

The prior probability of each outcome (consequence severity level *k* (*k* = 1, 2, 3, 4, 5 and 6), denoted by *P*(*C*_*k*_), is given as:

1 $$\begin{aligned} P\left( {C_{k} } \right) = &\Pi \, X_{i}^{\theta ,I,k} \left( {1 - x_{i} } \right)_{i,k}^{1 - \theta } \\ & j \in {\text{SB}}_{k} \\ \end{aligned}$$

where SB_*k*_ denotes the safety barrier associated with the level *k* and *θ*_*i*,*k*_ = 1 if the level *k* failure passes the down-branch (failure) of safety barrier *i*; *θ*_*i*,*k*_ = 0 if the level *k* failure passes the up-branch (success) of safety barrier *i*. Table 2 illustrates prior probabilities of consequence occurrence.

**Table 2:** Prior estimates of occurrences of each consequence

Consequences (*C*_*k*_)	Occurrence probability *P*(*C*_*k*_)
*C*_1_ (Safe)	0.8
*C*_2_ (Near miss)	0.19
*C*_3_ (Mishap)	9.5 × 10^−3^
*C*_4_ (Incident)	4.5 × 10^−4^
*C*_5_ (Accident)	4.5 × 10^−5^
*C*_6_ Catastrophe	5.0 × 10^−6^

### Modeling prediction and updating

Conditional or marginal probability approaches are widely utilized in classical accident modeling and risk assessment. These approaches are inaccurate predictors for a wide range of operating conditions (Tesfatsion 2015). However, the SMART approach is predicated on a rational prediction model attempting to ensure a more accurate predictive model for TENORM occupational exposure and associated risk by considering the two adjoined events (safety barriers failures and abnormal events) rather than a single event (abnormal events). Therefore, having a more accurate predictive model will enhance the safety system improvement decisions accuracy. Mathematically, the rational prediction model is presented as follows:

$$\begin{aligned} & P \, \left( {\text{data}} \right) \, = \, P \, \left( {{\text{data }}| \, X_{i} } \right) \\ & P \, \left( {\text{data}} \right) \, = \, P \, \left( {{\text{data }}|{\text{True}}} \right) \\ & P \, \left( {X_{i} } \right) \, = {{\left| {\left\{ {x: \, X_{i} \left( x \right)} \right\}} \right|} \mathord{\left/ {\vphantom {{\left| {\left\{ {x: \, X_{i} \left( x \right)} \right\}} \right|} {\left| {x:{\text{ true}}} \right|}}} \right. \kern-0pt} {\left| {x:{\text{ true}}} \right|}} \\ \end{aligned}$$

Then conditional probability expressed as:

$$P \, \left( {{\text{data }}| \, X_{i} } \right) \, = \, \left| {\left\{ {x: \, X_{i} \left( x \right)\;{\text{and}}\;{\text{data }}\left( X \right)} \right\}} \right|/ \, \left| {x: \, X_{i} \left( x \right)} \right|$$

Finally, the joint probability of this model expressed as:

$$\begin{aligned} P \, \left( {X_{i} \;{\text{and}}\;{\text{data}}} \right) \, = & \, {{\left| {\left\{ {x: \, X_{i} \left( x \right)\;{\text{and}}\;{\text{data}}\left( x \right)} \right\}} \right|} \mathord{\left/ {\vphantom {{\left| {\left\{ {x: \, X_{i} \left( x \right)\;{\text{and}}\;{\text{data}}\left( x \right)} \right\}} \right|} {\left| {x:{\text{ true}}} \right|}}} \right. \kern-0pt} {\left| {x:{\text{ true}}} \right|}} \\ & = {{\left| {\left\{ {x \, : \, X_{i} \left( x \right)\;{\text{and}}\;{\text{data }}\left( x \right)} \right\} \, } \right| \, \left| {\{ \left\{ {x \, : \, X_{i} \left( x \right)} \right\}} \right|} \mathord{\left/ {\vphantom {{\left| {\left\{ {x \, : \, X_{i} \left( x \right)\;{\text{and}}\;{\text{data }}\left( x \right)} \right\} \, } \right| \, \left| {\{ \left\{ {x \, : \, X_{i} \left( x \right)} \right\}} \right|} {\left( {\left\{ {x \, : \, X_{i} \left( x \right)} \right\}\left| \, \right|x \, :{\text{ true}}|} \right)}}} \right. \kern-0pt} {\left( {\left\{ {x \, : \, X_{i} \left( x \right)} \right\}\left| \, \right|x \, :{\text{ true}}|} \right)}} \\ & = \, P \, \left( {X_{i} } \right) \, P\left( {{\text{data }}| \, X_{i} } \right) \\ \end{aligned}$$

Using symmetry, this equation can be written as Bayes law as expressed in Eq. (2) (which is the base of this model) to estimate the likelihood and update failure probability of safety barriers in the next time interval (*t* + 1)

2 $$P \, \left( {X_{i} \;{\text{and}}\;{\text{data}}} \right) = P({\text{data}}|X_{i} ) \, *P(X_{i} )$$

where

- *P*(*X*_*i*_ and data) is the joint probability of two events (failure of safety barrier will occur first, and then abnormal event will take place and vice versa).
- *P*(data |*X*_*i*_) is the occurrence of abnormal events “data” given failures of safety barriers “*X*_*i*_” have occurred.
- *P*(*X*_*i*_) is the prior failure probabilities of safety barriers “*X*_*i*_”.

#### Failure probability estimation

The first step in the predictive model is to estimate the failure probability of the safety barriers for next time interval to prevent TENORM occupational exposure in oil and gas industry. Therefore, cumulative abnormal event data are a necessity to estimate the failure probability. These data were assumed with the consensus of technical experts according to the safety barriers failures to reflect the actuality of TENORM occupational exposure for ten years for workers involved in different oilfield oil and gas activities. Cumulative abnormal event data are shown in Table 3. The probabilities (Table 4) of precursors to abnormal events were computed based on the data provided in Table 3.

**Table 3:** Cumulative precursor data of abnormal events of TENORM exposure in oil and gas industry over 10 years

Years	*C*_1_ Safe	*C*_2_ Near miss	*C*_3_ Mishap	*C*_4_ Incident	*C*_5_ Accident	*C*_6_ Catastrophe
1	28	30	10	6	3	1
2	36	40	15	9	7	2
3	44	48	17	12	9	3
4	47	55	19	13	11	4
5	50	65	25	16	14	6
6	47	82	33	20	15	8
7	55	89	42	30	27	15
8	62	100	53	42	39	25
9	74	109	60	45	43	38
10	80	114	65	60	67	87
Total	523	732	339	253	235	189

**Table 4:** Probabilities of abnormal events precursor data of TENORM exposure in oil and gas industry over 10 years *P*(data|*X*_*i*_)

Years	*C*_1_ Safe	*C*_2_ Near miss	*C*_3_ Mishap	*C*_4_ Incident	*C*_5_ Accident	*C*_6_ Catastrophe
1	0.359	0.385	0.128	0.077	0.038	0.013
2	0.330	0.367	0.138	0.083	0.064	0.018
3	0.331	0.361	0.128	0.090	0.068	0.023
4	0.315	0.369	0.128	0.087	0.074	0.027
5	0.284	0.369	0.142	0.091	0.080	0.034
6	0.229	0.400	0.161	0.098	0.073	0.039
7	0.213	0.345	0.163	0.116	0.105	0.058
8	0.193	0.312	0.165	0.131	0.121	0.078
9	0.201	0.295	0.163	0.122	0.117	0.103
10	0.169	0.241	0.137	0.127	0.142	0.184

According to rational theory, the SMART approach considers the joint probability of occurrence of both events *P*(*X*_*i*_ and data) as a basis for the ensuing prediction of failure probability that are presented in Table 5.

**Table 5:** Rational probabilities of precursors of abnormal events of TENORM exposure in oil and gas industry over 10 years *P*(*X*_*i*_ and data)

Years	*C*_1_ Safe	*C*_2_ Near miss	*C*_3_ Mishap	*C*_4_ Incident	*C*_5_ Accident	*C*_6_ Catastrophe
1	0.187	0.077	0.006	0.004	0.004	0.001
2	0.172	0.073	0.007	0.004	0.006	0.002
3	0.172	0.072	0.006	0.005	0.007	0.002
4	0.164	0.074	0.006	0.004	0.007	0.003
5	0.148	0.074	0.014	0.005	0.008	0.003
6	0.119	0.080	0.008	0.005	0.007	0.004
7	0.111	0.069	0.008	0.006	0.010	0.006
8	0.100	0.062	0.008	0.007	0.012	0.008
9	0.104	0.059	0.008	0.006	0.012	0.010
10	0.088	0.048	0.007	0.006	0.014	0.018

Rational cumulative precursor data *P*(*X*_*i*_ and data) were then simulated using Monte Carlo simulation, where the objective was to simulate events of an identified period (*t* = 10 years) in an existing scenario for one thousand cycles to model all possible accidents processes and their causative behaviors based on the safety barriers performance as well as to determine how random variation and associated errors affect the modeled parametric system uncertainty and performance. The cumulative precursor data *P*(*X*_*i*_ and data) were defined as input for the parametric model for simulation and is denoted by *f*{(*X*_1_ and data), (*X*_2_ and data),…, (*X*_*i*_ and data)}. The probability distribution of the defined parametric model was utilized to generate another set of random inputs. These newly generated inputs were then evaluated and the same process was repeated for one thousand runs so that these data best-matched with the other data, or best-represented the current knowledge state, and are denoted by {(*X*_*i*_ and data)_1_, (*X*_*i*_ and data)_2_,…, (*X*_*i*_ and data)_*q*_}. Table 6 illustrates the improved quality of the cumulative precursor data of abnormal events extracted randomly from the simulated data.

**Table 6:** Cumulative precursor data of abnormal events simulated over ten years of TENORM occupational exposure *P*(*X*_*i*_ and data)

Years	*C*_1_ Safe	*C*_2_ Near miss	*C*_3_ Mishap	*C*_4_ Incident	*C*_5_ Accident	*C*_6_ Catastrophe
1	0.184	0.075	0.006	0.004	0.004	0.001
2	0.169	0.072	0.007	0.004	0.006	0.002
3	0.170	0.070	0.006	0.004	0.007	0.002
4	0.161	0.071	0.006	0.004	0.007	0.003
5	0.144	0.071	0.009	0.004	0.008	0.003
6	0.117	0.078	0.008	0.005	0.007	0.004
7	0.108	0.065	0.008	0.006	0.010	0.006
8	0.100	0.060	0.008	0.006	0.012	0.008
9	0.102	0.057	0.008	0.006	0.011	0.010
10	0.083	0.045	0.007	0.006	0.014	0.018

The generated data then were used to calculate the likelihood failure probability of safety barrier in the next time interval of ten years using Eq. (3):

3 $$\begin{aligned} & p\left( {{\text{data}}|xi} \right) \, = \, \left[ {N_{F,I} | \, \left( {N_{F,i} + \, N_{S,i} } \right)} \right] \\ & N_{S,I} = \, N_{C,k} ,\;{\text{for}}\;k \, = \, i \\ & N_{F,i} = \sum N_{c,k,} \;{\text{and}}\; \, k \, > \, i; \\ & \qquad \qquad i \, = \, 1, \, 2, \, 3, \, 4\;{\text{and}}\;k \, = \, 1, \, 2, \, 3, \, 4, \, 5 \\ \end{aligned}$$

where *N*_*c*,*k*_ is the number of abnormal events of consequence *k*th level, and *N*_*S*,*i*_ and *N*_*F*,*i*_ are the number of successes and failures for the *i*th barrier.

The failure probabilities for all safety barriers are listed in Table 7.

**Table 7:** Likelihood failure probabilities for all safety barriers *P*(*X*_*i*_ and data)

Years	EDSPB	IISPB	PPE&EDSPB	EMSPB	M&0 PB
1	0.328	0.164	0.577	0.574	0.259
2	0.349	0.205	0.645	0.667	0.226
3	0.344	0.215	0.681	0.670	0.255
4	0.361	0.219	0.694	0.704	0.272
5	0.398	0.256	0.631	0.720	0.305
6	0.464	0.231	0.666	0.705	0.353
7	0.467	0.312	0.729	0.744	0.362
8	0.485	0.362	0.762	0.757	0.394
9	0.475	0.383	0.780	0.779	0.470
10	0.519	0.497	0.854	0.837	0.569

#### Safety barriers failure probability update

The Bayesian updating mechanism was then utilized to update the safety barriers likelihood of failure probability over the following ten years when new types of evidence arise or changes occur in oil and gas processing. Thus, updated failure probabilities uncover the consequence occurrence probabilities, which were updated using event tree analysis. According to rational theory, the failure probabilities of a given safety barrier *X*_*i*_ are affected by a combination of latent or physical and dependent or independent random variables. These variables are considered as new evidence and therefore are added to the predictive and updating SMART model using the Bayesian updating theorem (Bedford and Cooke 2001) as per Eq. (4) as follows.

4 $$P\left( {X_{i} |{\text{data}}} \right) \, = \, {{\left[ {P\left( {{\text{data}}|X_{i} } \right) \, p\left( {X_{i} } \right)} \right]} \mathord{\left/ {\vphantom {{\left[ {P\left( {{\text{data}}|X_{i} } \right) \, p\left( {X_{i} } \right)} \right]} {\varSigma \left[ {P\left( {{\text{data}}|X_{i} } \right) \, P\left( {X_{i} } \right)} \right]}}} \right. \kern-0pt} {\varSigma \left[ {P\left( {{\text{data}}|X_{i} } \right) \, P\left( {X_{i} } \right)} \right]}}$$

where *P*(*X*_*i*_|data) is the posterior failure probability of safety barrier, *P*(data| *X*_*i*_) is the likelihood failure probability of safety barrier, *p*(xi) is the prior failure probability of safety barrier, data are the new information or evidences arrived and Σ [*P*(data|*X*_*i*_) *P*(*X*_*i*_)] is the normalizing factor.

**Fig. 4:**
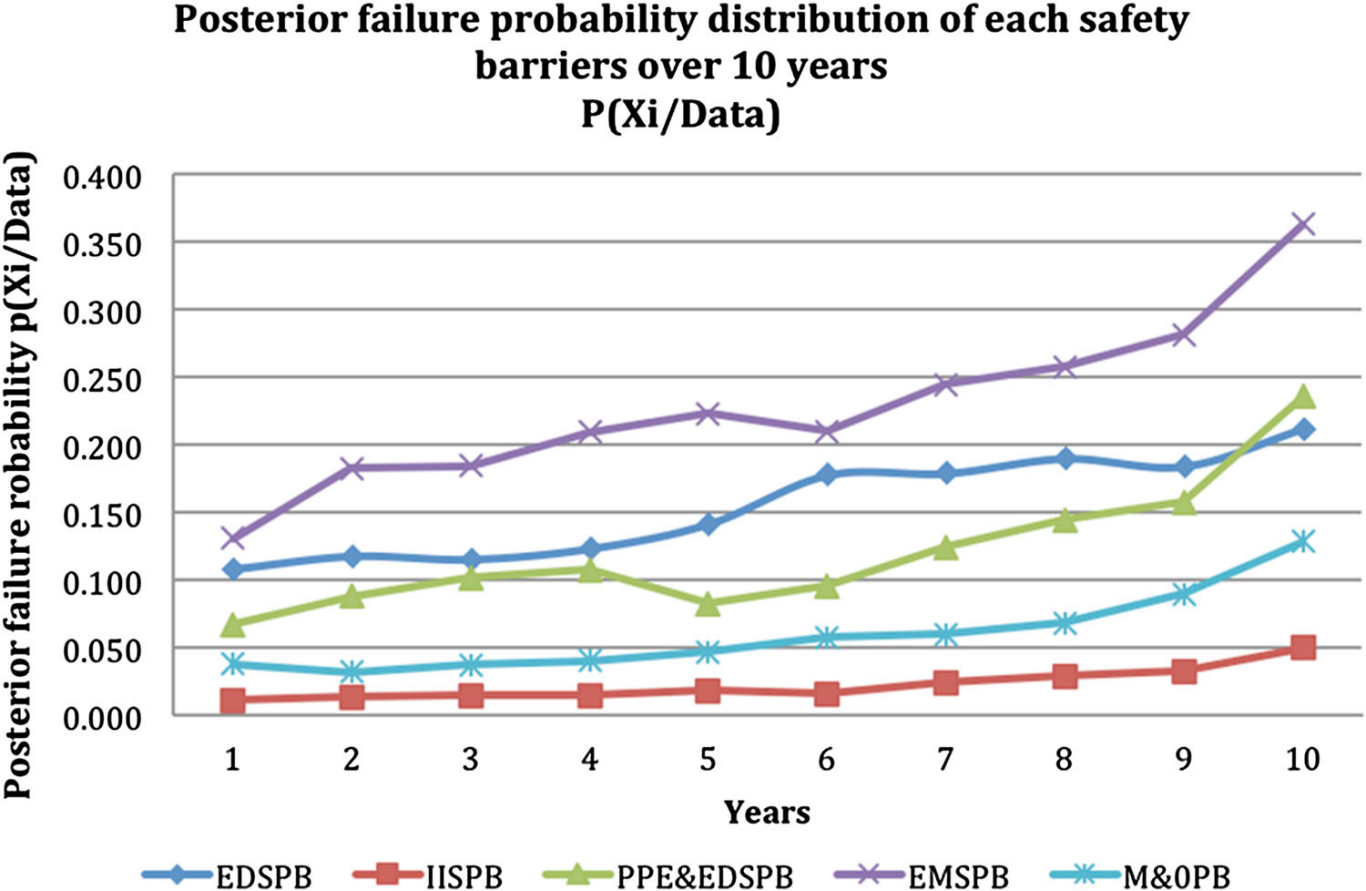
Posterior failure probability distribution of safety barriers failure over 10 years

Table 8 and Fig. 4 illustrate the updated failure probability for safety barriers over ten years and updated based on the arrival of new evidences that contributed into the failure probability of safety barriers.

**Table 8:** Posterior failure probability data for safety barriers performance updated over 10 years *P*(*X*_*i*_|data)

Years	EDSPB	IISPB	PPE&EDSPB	EMSPB	M&OPB
1	0.109	0.010	0.067	0.130	0.037
2	0.118	0.013	0.087	0.182	0.031
3	0.116	0.014	0.101	0.184	0.037
4	0.124	0.015	0.107	0.209	0.040
5	0.142	0.018	0.083	0.223	0.047
6	0.178	0.016	0.095	0.210	0.057
7	0.179	0.023	0.124	0.244	0.059
8	0.190	0.029	0.144	0.257	0.067
9	0.185	0.032	0.157	0.281	0.090
10	0.212	0.049	0.235	0.363	0.128

#### Consequence occurrence probability update

**Fig. 5:**
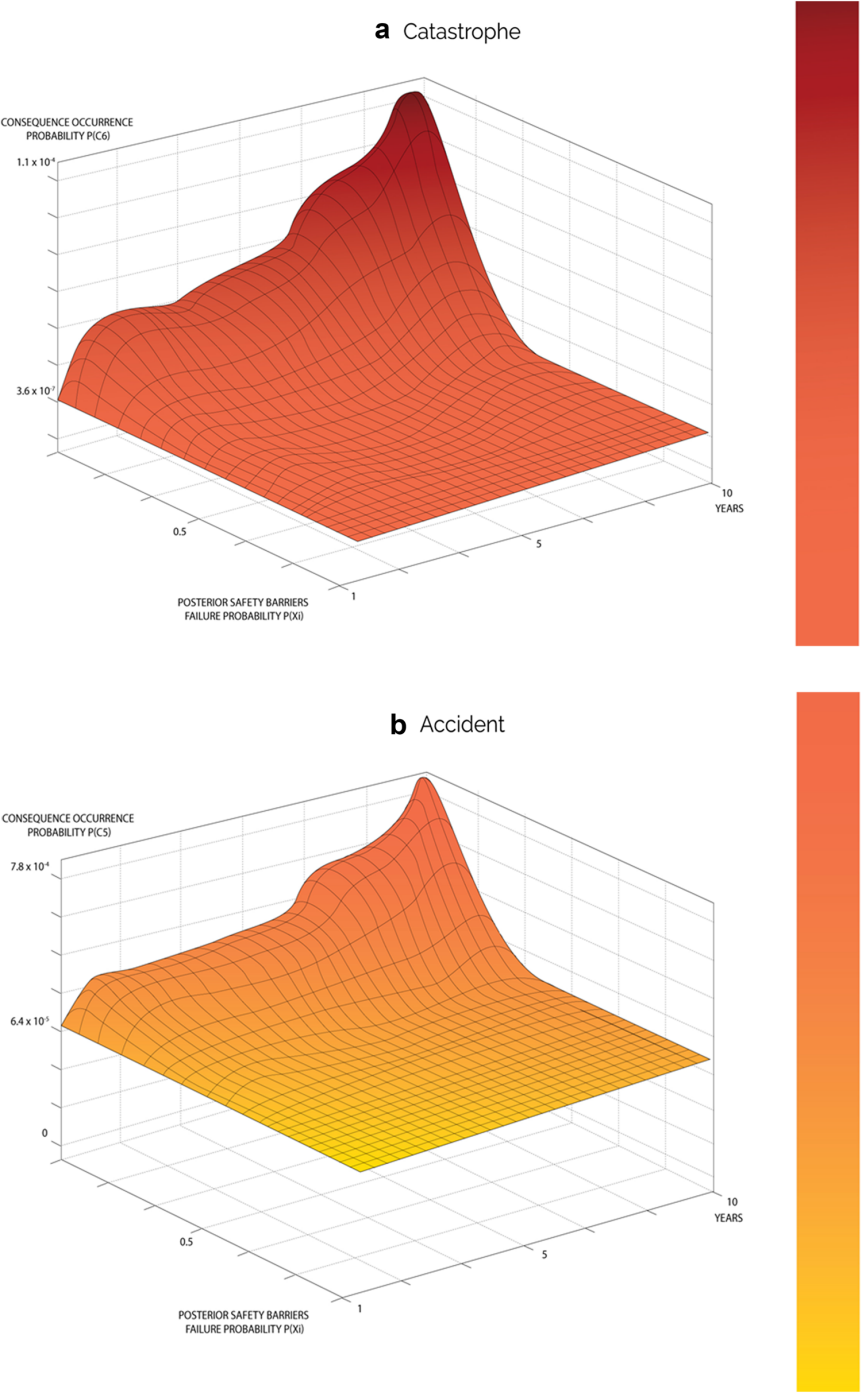

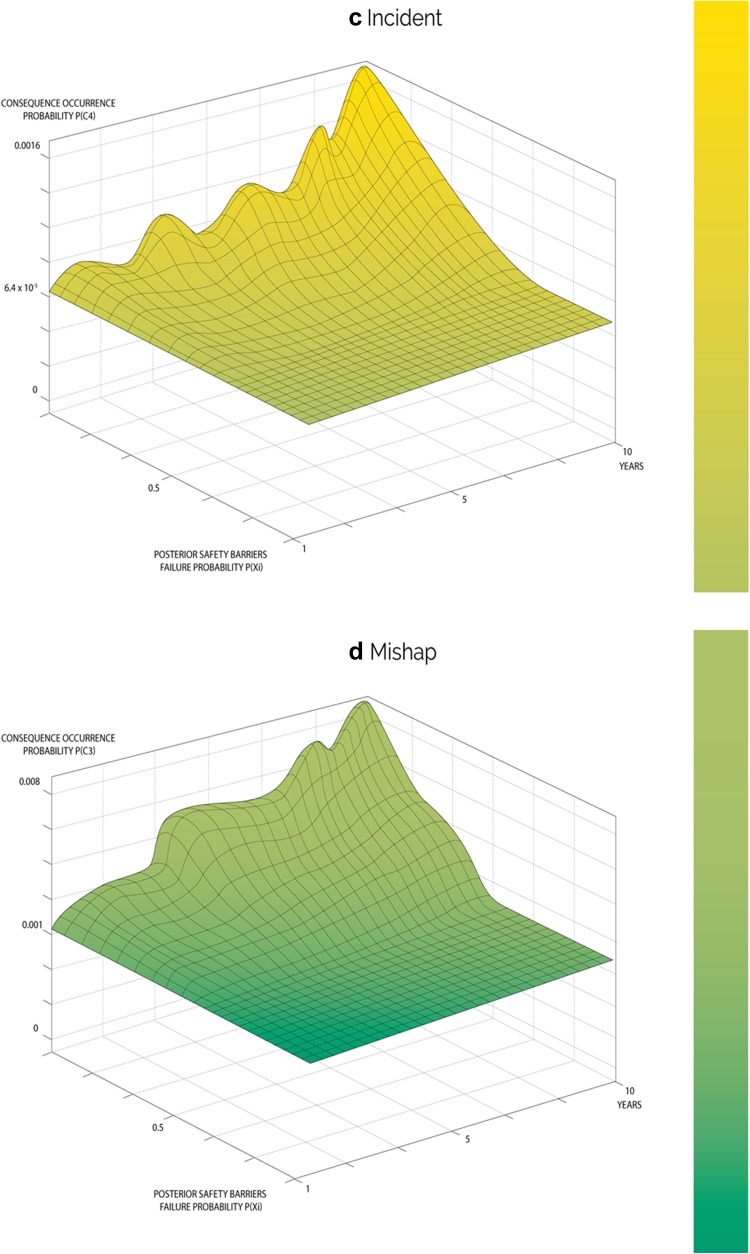

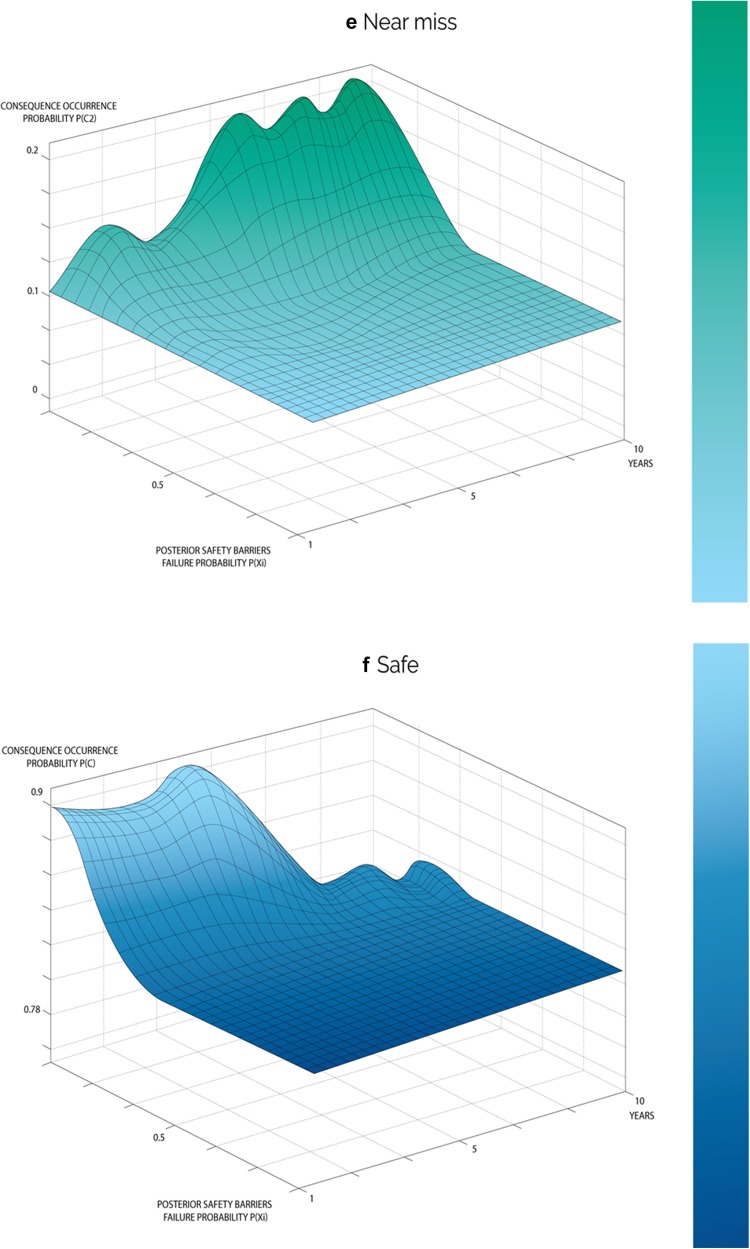
Overall variation of updated consequence occurrence probability distributions over a period of 10 years for range of events

The updated failure probabilities of the safety barriers in this model were utilized to estimate occurrence probabilities for each severity level. These probabilities were then fed into relevant branches of the event tree shown in Fig. 1, and Eq. (1) is utilized to estimate the posterior occurrence probabilities of each severity level over the ten years as shown in Fig. 5. Table 9 illustrates posterior probabilities of consequence occurrence in year ten.

**Table 9:** Posterior estimate of occurrence probability of each consequence in year 10

Consequences (*C*_*k*_)	Occurrence probability *P*(*C*_*k*_)
*C*_1_ (Safe)	0.788
*C*_2_ (Near miss)	0.201
*C*_3_ (Mishap)	8 × 10^−3^
*C*_4_ (Incident)	1.6 × 10^−3^
*C*_5_ (Accident)	7.8 × 10^−4^
*C*_6_ Catastrophe	1.1 × 10^−4^

## Analysis and discussions

The dynamic TENORM occupational exposure modeling risk was based on the performance of five identified sequential barriers and their sub-elements that are mostly absent in many oilfields. These safety barriers are the Early Detection Safety Prevention Barrier (EDSPB); Isolation Integrity Safety Prevention Barrier (IISPB); Personal Protection Equipment and Exposure Duration Safety Prevention Barrier (PPE&EDSPB); Emergency Management Safety Prevention Barrier (EMSPB) and Management and Organization Safety Prevention Barrier (M&OSPB). To test the model validity, a quantitative assessment was performed using the SMART methodology coupled with a probabilistic approach. Model validation was based on three main phases comprised of safety barriers analyses and evaluation, model prediction and updating and consequence occurrence probability updating.

According to the prior results, the consequences of higher severity have low probabilities of occurrence, which is obvious in events of catastrophe and accident. On the other hand, the consequences of lower severity have higher probabilities, such as safe events. For example, the probability of maintaining a safe system was 0.8, whereas the estimated probability of accident and catastrophic cancer fatality were very low 4.5 × 10^−5^ and 5 × 10^−6^, respectively. Based on the initial knowledge, it has been found that the probabilities of occurrence of other severity levels, such as near misses, mishaps and incidents gradually increased from 0.107 to 0.201, 0.001 to 0.008, and 6.4 × 10^−5^ to 0.002, respectively, as the system started to degrade. The obtained results from this model provided both qualitative and quantitative information about TENORM occupational exposure risk in the oil and gas industry. These results indicated that the proposed model is applicable to practical applications with the occurrence of a safe mode higher than fatal cancer causing event(s) (according to general medical radiological cancer data).

The rational prediction and Bayesian updating theorem adopted in the second phase of the SMART approach were utilized to predict the failure likelihood and update the prior failure probabilities of the identified safety barriers over the ten-year period. The prediction attempted to present a better visualization of the safety performance in ten-year time so that appropriate decisions can be made. As shown in Fig. 4, Bayesian posterior probability values for the safety barrier failures have drastically increased as a result of system degradation within the 10-year period. This degradation could be attributed to many reasons, the most important being a dearth of dynamic and quantitative radiological risk assessment studies related to TENORM risks in the oil and gas Industry. In addition, some of the legislation and TENORM-producing industries are reluctant to admit the presence of radiological risks in its operation despite avoiding any association with the word “nuclear” (ALNabhani et al. 2016a). Some industries consider that exposure to TENORM at a low dose is safe, while the medical community considering it unsafe according to their epidemiological studies (ALNabhani et al. 2016b). Furthermore, the implementation of cost-cutting plans by some industries may inhibit safety barriers improvement. Accordingly, the system will continue to degrade.

 The posterior failure probabilities of safety barriers were utilized in the third phase and were fed into event tree branches to estimate the updated occurrence probabilities of consequences. The results demonstrated system degradation cause the end-state probability (consequence occurrences probability) to change dramatically over the ten-year period. Prior probability of occurrence of the safe (*C*_1_) condition being high, its posterior probability was gradually reduced from 0.89 to 0.79 as time increased, as illustrated in Fig. 5f. This sharp drop raises concerns for its implication that the industry should be able to prevent such system degradation at early stages if the identified safety barriers were for early-stage activities. For instance, if an early detection prevention barrier was in place it will allow the industry to predict the presence of TONERM in their oilfield and well holdings at early stages by correlating logging data that contains radioactivity data, which are in fact utilized as an indicator of the presence of oil and gas of targeted pay zone formation (ALNabhani et al. 2015), and therefore, appropriate safety precautions can be taken. As a consequence of the safe mode deficiency, posterior probabilities of occurrence of incidents, accidents and catastrophes continued to drastically increase overtime, as shown in Fig. 5a–c respectively. The continual drastic increase could be attributed to failure of the subsequent safety barriers. If the first safety barrier failed, TENORM would then be brought up from the rock reservoir that hold oil and gas in their matrix, along with oil and gas extraction and production activities, and continue to flow from the drilled wells to gathering and production stations and finally to the refinery via well completion equipment, flow lines and associated equipment (Holland 1998; Jonkers et al. 1997; Wilson and Scott 1992; Hamlat et al. 2001; Abdel-Sabour 2014). This equipment is unfortunately not radiologically insulated or designed to prevent gamma radiation emitted by TENORM passing through or in their scale depositions. As a result of the failure of second safety barrier, many workers involved in oil and gas extraction and production activities are at risk of being exposed to different radiation levels. In particular, current standard personal protective equipment (third safety prevention barrier) is not designed to handle accidental exposure to any radiation, let alone for nearly constant daily, weekly and even year long exposure times. The risk of exposure to radiation doses at elevated levels may develop into fatal cancer within 10 years of continuous and cumulative exposure. According to the model results, the posterior probability of a fatal cancer catastrophe (*C*_6_) improved greatly during the ten years of continuous exposure; however, it has a sharp increasing tendency in probability from 3.6 × 10^−07^ to 1.1 × 10^−4^ as shown in Fig. 5a that is almost a 3000-fold increase, which raises serious concerns. Most importantly, some safety barriers such as the Emergency Management Safety Prevention Barrier (EMSPB) and Management and the Organization Safety Prevention Barrier (M&OSPB) can interact and intervene with the whole safety system at any stage during operation and their interaction can promote safety strategies, or in the opposite manner, weaken the safety system based on the management’s behavior and their awareness of safety importance. This can be clearly observed when looking to the posterior occurrence probabilities of near miss (*C*_2_), mishap (*C*_3_), incident (*C*_4_) and accident (*C*_5_) that frequently occur in the industry. Figure 5b–e shows a fluctuating trend between steadily rising and sudden sharp increases overtime. The reason behind the fluctuation is that only in the events of observing radiation are the preventive measures applied based on its causal factors and occurrence frequency and, therefore, prove this phenomenon. However, over extended time periods, the system re-exhibits performance impairment.

## Conclusions

TENORM is a potentially serious environmental and occupational risk in oil and gas operations. To assess radiation exposure risk to workers, a new methodology of dynamic modeling scenario-based risk assessment was proposed. This model was based on SMART approach that integrates the SHIPP methodology and rational theory. This approach provided a systematic and comprehensive risk assessment framework based on safety barrier performance evaluation and analysis. Five important safety barriers were identified and are considered to provide workers sufficient protection from radiation exposure during oil and gas extraction and production activities. The SMART approach provides a systematic framework for modeling, predicting, updating and managing the TENORM exposure risk during oil and gas production. This paper is considered as the first fruits in the radiological occupational exposure risk assessment area of the oil and gas industry to quantify TENORM risk and interpret it with safety barrier performance. Based on the results, it is apparent there is a need to develop appropriate safety measures for protecting against radiation exposure during extraction and production of oil and gas and to find an effective scientifically based solution to minimize the large radiological waste volume created during production that can result in serious radiological issues for workers, the public and the environment. The future works yet to be explored according to the SMART approach process flowchart are the estimation of the TENORM economic risk, and to establish a successful and thorough TENORM management system.
